# Cardiovascular Effects of Switching From Tobacco Cigarettes to Electronic Cigarettes

**DOI:** 10.1016/j.jacc.2019.09.067

**Published:** 2019-12-24

**Authors:** Jacob George, Muhammad Hussain, Thenmalar Vadiveloo, Sheila Ireland, Pippa Hopkinson, Allan D. Struthers, Peter T. Donnan, Faisel Khan, Chim C. Lang

**Affiliations:** a Division of Molecular and Clinical Medicine, University of Dundee, Ninewells Hospital and Medical School, Dundee, United Kingdom; b Population Health and Genomics Division, University of Dundee, Ninewells Hospital and Medical School, Dundee, United Kingdom; c Division of Systems Medicine, University of Dundee, Ninewells Hospital and Medical School, Dundee, United Kingdom

**Keywords:** electronic cigarette, endothelial function, vascular stiffness, CI, confidence interval, CO, carbon monoxide, CV, cardiovascular, EC, electronic cigarette, FMD, flow-mediated dilation, OR, odds ratio, PWV, pulse wave velocity, TC, tobacco cigarette

## Abstract

**Background:**

E-cigarette (EC) use is increasing exponentially worldwide. The early cardiovascular effects of switching from tobacco cigarettes (TC) to EC in chronic smokers is unknown. Meta-analysis of flow-mediated dilation (FMD) studies indicate 13% lower pooled, adjusted relative risks of cardiovascular events with every 1% improvement in FMD.

**Objectives:**

This study sought to determine the early vascular impact of switching from TC to EC in chronic smokers.

**Methods:**

The authors conducted a prospective, randomized control trial with a parallel nonrandomized preference cohort and blinded endpoint of smokers ≥18 years of age who had smoked ≥15 cigarettes/day for ≥2 years and were free from established cardiovascular disease. Participants were randomized to EC with nicotine or EC without nicotine for 1 month. Those unwilling to quit continued with TC in a parallel preference arm. A propensity score analysis was done to adjust for differences between the randomized and preference arms. Vascular function was assessed by FMD and pulse wave velocity. Compliance with EC was measured by carbon monoxide levels.

**Results:**

Within 1 month of switching from TC to EC, there was a significant improvement in endothelial function (linear trend β = 0.73%; 95% confidence interval [CI]: 0.41 to 1.05; p < 0.0001; TC vs. EC combined: 1.49%; 95% CI: 0.93 to 2.04; p < 0.0001) and vascular stiffness (−0.529 m/s; 95% CI: −0.946 to −0.112; p = 0.014). Females benefited from switching more than males did in every between-group comparison. Those who complied best with EC switch demonstrated the largest improvement. There was no difference in vascular effects between EC with and without nicotine within the study timeframe.

**Conclusions:**

TC smokers, particularly females, demonstrate significant improvement in vascular health within 1 month of switching from TC to EC. Switching from TC to EC may be considered a harms reduction measure. (Vascular Effects of Regular Cigarettes Versus Electronic Cigarette Use [VESUVIUS]; NCT02878421; ISRCTN59133298)

Electronic cigarettes or E-cigarettes (EC) are gaining popularity worldwide as an alternative to smoking tobacco cigarettes (TC) with a 55% increase in users between 2013 and 2015 with growth in the United Kingdom occurring fastest in Europe (1). The prevalence of EC use in the United Kingdom and United States is around 6% (2), and 51% of users did so because they believed it to be less harmful than regular cigarettes (3). Observational data in the 2014 and 2016 U.S. National Health Interview Surveys revealed that although the risk of myocardial infarction remains higher with TC (odds ratio [OR]: 2.72; 95% confidence interval [CI]: 2.29 to 3.24), daily EC use was also associated with an increased myocardial infarction risk (OR: 1.79; 95% CI: 1.20 to 2.66) (4). Despite this, there remains little good quality evidence on the short- and long-term safety of these devices. Furthermore, conflicting advice from various public health bodies worldwide on the use of these devices has resulted in lack of clarity for policymakers as well as the public at large (5,6).

TC contain >7,000 chemicals, including exposing smokers to high levels of nicotine, carbon monoxide (CO), acrolein, and pro-oxidant compounds. Data from chemical analysis and toxicology studies suggest that exposure to toxic chemicals from EC is lower compared with exposure from TC (7,8). However, other studies have shown that there remains the presence of potentially harmful tobacco-specific alkaloids such as anabasine, myosmine, and β-nicotyrine in EC liquid cartridge samples tested (9). The impact of nicotine on vascular health is also unclear. Nicotine may accelerate the atherogenic process by binding to high-affinity nicotinic acetylcholine receptor cell surface receptors (10). However, longer-term nicotine use appears not to accelerate atherogenesis but may contribute to acute cardiovascular events in the presence of cardiovascular (CV) disease (11). The early vascular impact of switching from TC to EC--nicotine versus EC- nicotine-free is not known.

Endothelial dysfunction is the earliest detectable change in vascular health, and, importantly, it has consistently been shown to be associated with CV risk and long-term outcomes (12,13). We measured endothelial function using flow-mediated dilatation (FMD) and arterial stiffness by pulse wave velocity (PWV), 2 validated and independent predictors of CV risk above and beyond traditional risk factors (14,15). We conducted the current trial to address specific questions on the early CV effects of switching from TC to EC and the impact of nicotine itself on any early vascular changes that might be seen.

## Methods

The VESUVIUS (Vascular Effects of Regular Cigarettes Versus Electronic Cigarette Use) trial (NCT02878421) was a prospective, randomized controlled trial with a parallel, nonrandomized preference cohort and blinded endpoint of smokers ≥18 years of age who had smoked ≥15 cigarettes/day for at least 2 years; were free from established CV disease, diabetes, and chronic kidney disease; and were not on medication for those conditions. The trial was conducted between August 2016 and July 2018 in a single tertiary research center. Participants were recruited from local advertisements, smoking cessation databases, and visits to local businesses, as well as via the Scottish Primary Care Research Network. Consented participants who were willing to quit smoking were randomized to one of the EC arms in a 1:1 fashion using a centrally controlled web-based good clinical practices–compliant randomization system (TrusT, Health Informatics Centre, University of Dundee) to either: 1) EC containing 16 mg nicotine (Vapourlites Starter Kit with XR5 16 mg nicotine cartomizer; Vapourlites, Peterlee, United Kingdom); or 2) nicotine-free EC plus nicotine flavoring (Vapourlites Starter Kit with 0 mg nicotine cartomizer) because it was considered by the institutional ethics committee as ethically unacceptable to randomize those who were willing to quit smoking into a smoking arm. Those unwilling to consider quitting smoking continued in the parallel preference TC cohort. Participants in the TC arm continued their usual daily smoking habits and did not use EC for the 4-week period of the trial. The study was approved by the Tayside Research Ethics Committee and was carried out in accordance with the Declaration of Helsinki. Exhaled CO breath test was measured as an indicator of treatment allocation adherence to EC, as in previous trials (16), and was added to the primary model to assess the effect of adherence. Studies have previously shown that CO levels fall significantly when switching completely from TC to EC (17).

The primary efficacy endpoint was change in FMD among the TC group and the EC-nicotine and EC-nicotine-free groups.

### Flow-mediated dilation

Endothelial function was assessed by measuring FMD of the brachial artery using a Sequoia 512 (Siemens, Camberley, United Kingdom) and an 8-MHz linear array ultrasound probe as described previously (18). Patients fasted overnight and measurements were conducted at baseline and 1 month according to the International Brachial Artery Reactivity Task Force guidelines (19) by a single operator (M.H.) blinded to study allocation at a single site. All participants were required to refrain from smoking TC or EC for 4 h before each FMD test.

### Pulse wave velocity

Pulse wave velocity and augmentation index were measured at baseline and 1 month by a single operator (M.H.) blinded to study allocation. Measurements were recorded with a SphygmoCor (AtCor, Sydney, Australia) machine using a high-fidelity micromanometer.

### Biomarkers

We measured oxidized low-density lipoprotein, high-sensitivity C-reactive protein, tissue plasminogen activator, and platelet activation inhibitor-1 at baseline and at 1 month. All biomarkers were measured by enzyme-linked immunosorbent assay at the Immunoassay Biomarker Core Laboratory, University of Dundee.

### Outcomes

The primary outcome was defined as the change in FMD among the TC group and the EC-nicotine and EC-nicotine-free arms as a linear contrast. Secondary outcomes included change in FMD, PWV, augmentation index at 75 beats/min, heart rate, blood pressure, and biomarkers (oxidized low-density lipoprotein, high-sensitivity C-reactive protein, tissue plasminogen activator, and platelet activation inhibitor-1) for the TC, EC-nicotine, and EC-nicotine-free arms.

### Statistical analysis

The primary endpoint of change in brachial artery FMD is expressed as the maximum FMD percentage change from baseline. Using linear contrast tests, a sample size of 36 subjects in each group would have 80% power to detect an improvement in FMD of 2.0% and 1.0% in the EC-nicotine-free and EC-nicotine arms, respectively, compared with the TC group at 5% significance. In this explanatory trial, all dropouts were replaced to achieve 36 completed subjects in each group. The primary analysis was performed on a per-protocol basis.

Descriptive statistics in the form of mean ± SD for continuous variables and percentages and denominators for categorical variables are tabulated for baseline and at the 1-month visit. The dependent variable was assessed for approximation to a normal distribution and transformed if necessary. The FMD response relationship was assessed by a linear contrast test (TC, EC-nicotine, EC-nicotine-free) in a multiple linear regression on FMD at 4 weeks including the baseline FMD level and experimental group as covariates. The model also included the minimization variables: baseline age (≤40 years, >40 years); sex (male, female); and smoking pack-years (≤20 pack-years, >20 pack-years). Pre-specified subgroup analyses were completed by fitting the appropriate interaction term in the regression model and, if significant, outcomes were analyzed separately by level of subgroup. All comparisons were performed among treatment arms (TC vs. EC-nicotine vs. EC-nicotine-free) at the final visit (4 weeks) and adjusted for the baseline measure of the outcome.

As the parallel control arm expressed a preference to not be randomized, a propensity score was created with the binary outcome of randomized versus nonrandomized using logistic regression and subsequently used as an adjustment covariate in the regression models to allow for potential bias. Variables included in the propensity score included demographic data, blood pressure, CO levels, all measured biomarkers, FMD and vascular stiffness parameters, and smoking history (Online Table 1). All analyses were conducted in SAS version 9.4 (SAS Institute Inc., Cary, North Carolina).

## Results

**Figure 1:**
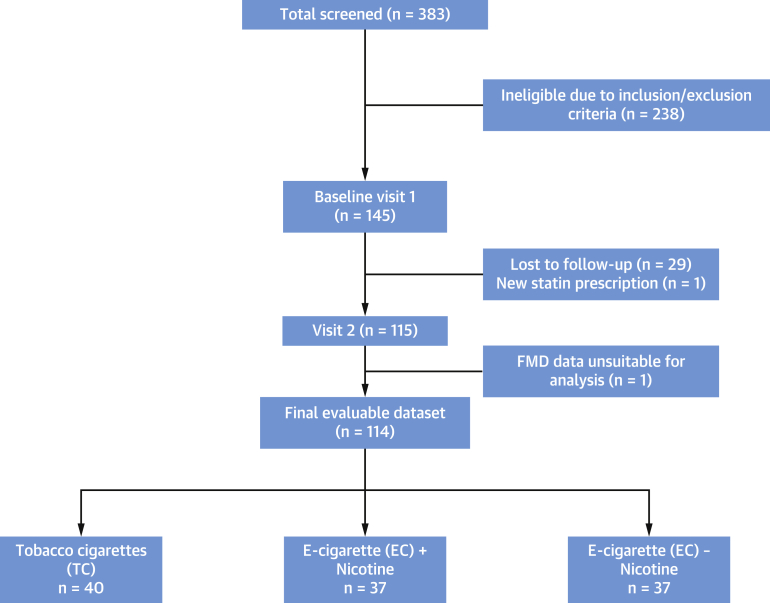
CONSORT Diagram Flow chart showing patient involvement in the study. CONSORT = Consolidated Standards of Reporting Trials; EC = electronic cigarettes; FMD = flow-mediated dilation; TC = tobacco cigarettes.

A total of 145 patients were recruited into the trial (Figure 1). A final number of 114 patients (40 TC, 37 EC-nicotine, 37 EC-nicotine-free) completed both visits. Baseline demographic data and smoking history among the 3 arms were comparable and are shown in Table 1. There were no serious adverse events reported during the trial.

**Table 1:** Demography of the Evaluable Dataset by Study Arm

	TC (n = 40, 35.0%)	EC-Nicotine (n = 37, 32.5%)	EC-Nicotine-Free (n = 37, 32.5%)
Male	13 (32.5)	14 (37.8)	12 (33.4)
Age, yrs	44.2 (40.4–47.9)	48.0 (44.7–51.3)	48.4 (43.5–53.3)
Weekly alcohol intake, U	0.0 [0.0–11.0]	0.0 [0.0–10.0]	4.0 [0.0–12.0]
BMI	26.7 (25.0–28.5)	28.1 (25.8–30.4)	27.1 (25.4–28.8)
Employment status			
FT	23 (57.5)	14 (37.8)	16 (43.2)
PT	3 (7.5)	5 (13.5)	7 (18.9)
Unemployed	7 (17.5)	10 (27.0)	7 (18.9)
Other	7 (17.5)	8 (21.6)	7 (18.9)
CO, ppm	12.0 [7.3–20.8]	12.0 [7.5–16.0]	11.0 [7.0–14.0]
CO% COHb	2.6 [1.8–4.0]	2.6 [1.9–3.2]	2.4 [1.8–2.9]
Age started smoking, yrs	15.0 [13.0–16.5]	14.0 [13.0–16.0]	16.0 [13.0–18.0]
Cigarettes per day	20 [15–20]	18 [15–20]	18 [15–20]
Years smoked	29.0 [19.5–36.5]	36.0 [25.0–41.0]	32.0 [22.0–40.0]
Pack-year history	25.4 [15.5–36.5]	33.3 [21.8–44.0]	27 [19.9–36.8]
Parents smoked			
No	8 (20.0)	6 (16.2)	10 (27.0)
Yes	32 (80.0)	31 (83.8)	27 (73.0)
Other smokers in the home			
0	23 (57.5)	24 (64.9)	29 (78.4)
1	15 (37.5)	13 (35.1)	8 (21.6)
2	2 (5.0)	0 (0.0)	0 (0.0)

Values are n (%), mean (95% confidence interval), or median [interquartile range]. Analysis of variance used for age, height, weight, BMI, systolic BP, diastolic BP and heart rate. Chi-square test used for categorical variables, sex, and employment status. Kruskal-Wallis test used for age started smoking, cigarettes per day, years smoked, pack-year history weekly alcohol intake, CO ppm, and CO% COHb.

BMI = body mass index; BP = blood pressure; CO = carbon monoxide; CO% COHb = percentage of CO in carboxyhemoglobin; EC = electronic cigarettes; FT = full time; ppm = parts per million; PT = part time; TC = total cigarettes.

### Primary outcome

The primary outcome of change in FMD of the brachial artery showed a significant trend in the difference among arms from TC to EC-nicotine to EC-nicotine-free (linear trend β for TC, EC-nicotine, EC-nicotine-free = 0.73%; 95% CI: 0.41 to 1.05; p < 0.0001). Within 1 month of switching from TC to EC, FMD significantly improved among TC and combined EC arms (1.49%; 95% CI: 0.93 to 2.04; p < 0.0001) and separately between TC and EC-nicotine and between TC and EC-nicotine-free (Table 2). There was no statistically significant difference in FMD change between the EC-nicotine and EC-nicotine-free arms (Table 2, Central Illustration).

**Table 2:** Regression Analysis of Outcomes for FMD—Linear Contrast With Higher Arm Less Nicotine

	Difference Between Arms in Change	p Value
Primary outcome*		
Change in FMD (+1 group, 1 = TC, 2 = EC-nicotine, 3 = EC-nicotine-free)	0.73 (0.41 to 1.05)	<0.0001
Secondary outcomes*		
Change in FMD, EC-nicotine-free vs. TC (ref)	1.52 (0.90 to 2.15)	<0.0001
Change in FMD, EC-nicotine vs. TC (ref)	1.44 (0.78 to 2.09)	<0.0001
Change in FMD, all EC vs. TC (ref)	1.49 (0.93 to 2.04)	<0.0001
Change in FMD, EC-nicotine-free vs. EC-nicotine (ref)	0.09 (−0.52 to 0.69)	0.78

Values are regression coefficient (95% CI).

CI = confidence interval; FMD = flow-mediated dilatation; ref = reference; other abbreviations as in Table 1.

∗ Adjusted for baseline of the outcome, baseline age (≤40 years, >40 years), sex (male, female), and smoking pack-years (≤20 pack-years, >20 pack-years).

**Central Illustration:**
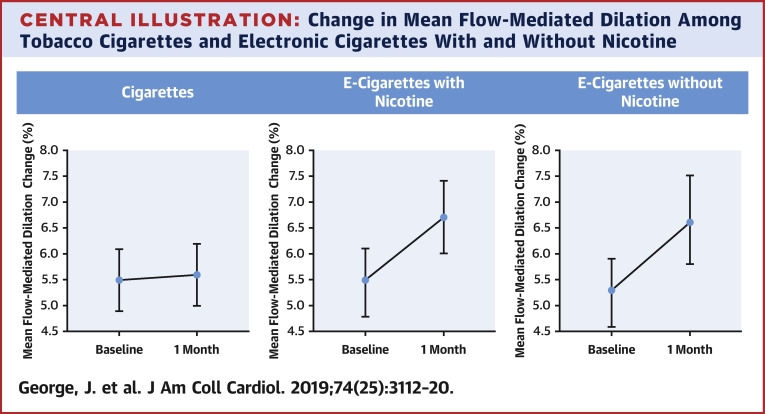
Change in Mean Flow-Mediated Dilation Among Tobacco Cigarettes and Electronic Cigarettes With and Without Nicotine Adjusted mean percentage change in forearm flow-mediated dilation with 95% confidence intervals for subjects on electronic cigarettes (EC), EC-nicotine, and EC-nicotine-free.

The interaction term between treatment and sex for the primary outcome of FMD change was statistically significant (p = 0.009), therefore the subgroup analyses was performed by sex. The improvement in FMD was seen in both males and females for TC versus EC comparisons but significantly greater improvement in vascular function was seen in females who switched from TC to EC (Table 3).

**Table 3:** Regression Analysis of FMD Primary Outcomes by Sex Subgroup—Linear Contrast With Higher Arm Less Nicotine

	Difference Between Arms in Change	p Value
Change in FMD (+1 group, 1 = TC, 2 = EC-nicotine, 3 = EC-nicotine-free)		
Male	0.213 (−0.248 to 0.675)	0.351
Female	1.049 (0.617 to 1.480)	<0.0001
Change in FMD, EC-nicotine-free vs. TC (ref)		
Male	0.448 (−0.451 to 1.347)	0.315
Female	2.183 (1.336 to 3.030)	<0.0001
Change in FMD, EC-nicotine vs. TC (ref)		
Male	0.822 (−0.067 to 1.710)	0.069
Female	1.824 (0.942 to 2.706)	<0.0001
Change in FMD, EC-nicotine-free vs. EC-nicotine (ref)		
Male	−0.374 (−1.239 to 0.492)	0.384
Female	0.359 (−0.449 to 1.167)	0.377

Values are regression coefficient (95% CI).

Adjusted for baseline of the outcome, baseline age (≤40 years, >40 years) and smoking pack-years (≤20 pack-years, >20 pack-years).

Abbreviations as in Tables 1 and 2.

As expected, exhaled CO levels were high at baseline and comparable among the 3 arms of the study (Table 1). However, at the end of study, those with the lowest tertile of CO (best compliance with EC and least dual use) had the greatest gain in vascular function improvement. In the lowest tertile of CO, once again, females who switched from TC to EC had a much greater gain in vascular function improvement than did males. Females who complied less well with allocated therapy (dual use with TC) at the middle and high CO tertiles, still benefited from switching to EC more than males did (Table 4). Data on noncompliant subjects are shown in Online Table 2.

**Table 4:** Change in FMD—Mean and 95% CI by CO Tertiles, Sex, and Group

CO Tertile	Sex	TC	EC-Nicotine	EC-Nicotine-Free
Low, 0–5 ppm	Male	0.28∗ [1]	1.23 (0.02 to 2.44) [6]	0.79 (0.38 to 1.21) [6]
	Female	0.29∗ [1]	1.58 (0.50 to 2.66) [12]	2.26 (1.31 to 3.21) [11]
	Both	0.29 (0.22 to 0.35)	1.46 (0.71 to 2.22)	1.74 (1.05 to 2.43)
Middle, 6–11 ppm	Male	0.17 (−0.57 to 0.91) [6]	0.81 (−5.39 to 7.00) [2]	−0.23 (−3.13 to 2.68) [3]
	Female	−0.64 (−1.76 to 0.47) [9]	0.87 (0.02 to 1.72) [7]	1.43 (0.71 to 2.15) [10]
	Both	−0.32 (−1.01 to 0.37)	0.86 (0.22 to 1.50)	1.05 (0.31 to 1.79)
High, 12–32 ppm	Male	0.43 (−0.40 to 1.25) [6]	0.83 (−0.40 to 2.07) [6]	0.51 (−3.81 to 4.83) [3]
	Female	0.16 (−0.30 to 0.62) [17]	1.74 (−0.77 to 4.25) [4]	1.55 (0.59 to 2.52) [4]
	Both	0.23 (−0.14 to 0.60)	1.20 (0.23 to 2.16)	1.11 (−0.03 to 2.24)

Values are mean (95% CI) [n].

Abbreviations as in Tables 1 and 2.

∗ 95 CI% not estimable.

Data from our lab for age- and sex-matched nonsmoking healthy volunteers indicate a mean FMD of 7.7%. To put this into context, over a 4-week switch, chronic smokers who switched from TC to EC-nicotine showed improved mean FMD from 5.5% to 6.7% and those who switched from TC to EC-nicotine-free showed improved mean FMD from 5.3% to 6.6%.

### Secondary outcomes

There was no significant trend in difference among the 3 arms for other secondary outcomes including PWV, heart rate, and biomarkers of inflammation and platelet reactivity (Table 5). However, the interaction terms between treatment and smoking pack-years were significant for PWV (p = 0.016) and heart rate (p = 0.003). Therefore, a subgroup analysis was done for these outcomes by smoking pack-years.

**Table 5:** Regression Analysis of Secondary Outcomes—Linear Contrast With Higher Arm Less Nicotine

	Difference Between Arms in Change	p Value
Carotid femoral pulse wave velocity	−0.167 (−0.402 to 0.069)	0.164
≤20 pack-years, n = 27	−0.471 (−0.834 to −0.107)	0.014
>20 pack-years, n = 70	0.031 (−0.271 to 0.332)	0.839
Heart rate	−1.190 (−3.050 to 0.670)	0.207
≤20 pack-years, n = 31	2.647 (0.278 to 5.016)	0.030
>20 pack-years, n = 82	−2.825 (−5.223 to −0.426)	0.022
Augmentation index,75 beats/min	0.112 (−1.833 to 2.058)	0.909
Oxidized LDL	−1.113 (−5.458 to 3.232)	0.612
High-sensitivity CRP∗	0.039 (−0.221 to 0.299)	0.769
Tissue plasminogen activator∗	−0.036 (−0.123 to 0.052)	0.425
Platelet activation inhibitor-1∗	−0.007 (−0.131 to 0.116)	0.906
Systolic blood pressure	−2.158 (−4.789 to 0.472)	0.107
Diastolic blood pressure	−1.126 (−2.624 to 0.372)	0.139

Values are regression coefficient (95% CI). Change in parameters adjusted for baseline of the outcome, baseline age (≤40 years, >40 years), sex (male, female), and smoking pack-years (≤20 pack-years, >20 pack-years).

CI = confidence interval; CRP = C-reactive protein; LDL = low-density lipoprotein.

∗ Log-transformed.

### Vascular stiffness and blood pressure

Smokers who smoked ≤20 pack-years also demonstrated an improvement in vascular stiffness within 1 month of switching from TC to EC with an improvement in PWV (−0.471 m/s; 95% CI: −0.834 to −0.107; p = 0.014), whereas those who smoked >20 pack-years showed no change within this time frame (Table 5). For the whole cohort, when both EC arms were combined, there was a significant improvement in PWV in this combined EC group compared with in the TC group (−0.529 m/s; 95% CI: −0.946 to −0.112; p = 0.014). When both EC groups were combined, there was a greater reduction in systolic blood pressure in the EC group than in the TC group, both in smokers of ≤20 pack-years (EC: −4.41 mm Hg; 95% CI: −7.91 to −0.91 vs. TC: −2.86 mm Hg; 95% CI: −8.09 to 2.38; p = 0.59) and >20 pack-years (EC: −7.75 mm Hg; 95% CI: −11.56 to −3.93 vs. TC: −1.37 mm Hg; 95% CI: −5.32 to 2.59; p = 0.04).

Using analysis of variance, there was a significant difference for the mean change of systolic blood pressure among the 3 arms: TC (−1.89 mm Hg; 95% CI: −4.91 to 1.14); EC-nicotine (−4.27 mm Hg; 95% CI: −7.73 to −0.81); EC-nicotine-free (−9.69 mm Hg; 95% CI: −14.67 to −4.71), p = 0.01. When adjusted for baseline variables, the trend remained toward lower systolic blood pressures among arms from TC to EC-nicotine to EC-nicotine-free but was not statistically significant (β = −2.2 mm Hg; 95% CI: −4.8 to 0.5; p = 0.11). The greatest difference in systolic blood pressure was seen in the TC versus EC-nicotine-free arms (−4.3 mm Hg; 95% CI: –9.6 to 1.0; p = 0.11) followed by TC versus EC-nicotine arms (−2.0 mm Hg; 95% CI: –7.6 to 3.5; p = 0.47).

### Heart rate

For smokers who smoked ≤20 pack-years (n = 31), resting heart rate significantly increased by 2.6 beats/min (95% CI: 0.3 to 5.0) for EC-nicotine compared with the rate for TC and increased by 5.2 beats/min (95% CI: 0.6 to 10.0) for EC-nicotine-free compared with the rate for TC (p = 0.03). However, for smokers who smoked >20 pack-years (n = 82), resting heart rate decreased by 2.8 beats/min (95% CI: −5.2 to −0.4) for EC-nicotine compared with the rate for TC and decreased further by 5.6 beats/min (95% CI: −10.4 to −0.8) for EC-nicotine-free compared with the rate for TC (p = 0.02).

## Discussion

The main findings from this present study are that within 1 month of switching from TC to EC, smokers demonstrate a significant improvement in vascular function. The data from this present trial on the early CV impact of switching from TC to EC has yielded several clinically important findings.

First, there is an early benefit to vascular function from switching from TC to EC. Within the switching time frame of 1 month, chronic smokers demonstrated significant improvements in vascular endothelial function. This is consistent with the recent review by Benowitz and Fraiman (20) that switching from TC to EC might result in overall benefit to public health. Previous meta-analysis of FMD studies have demonstrated that the pooled, adjusted relative risks of CV events was 13% lower with every 1% improvement in FMD (12). As we have demonstrated with age- and sex-matched healthy volunteer FMD data from our lab, otherwise healthy but chronic TC smokers who switched to EC improved their vascular function, approaching values seen in healthy nonsmokers. Within 1 month of switching from TC, we found a 1.5% improvement between TC and EC-nicotine-free arms, 1.4% improvement between TC and EC-nicotine arms, and a 1.5% improvement between TC and combined EC arms (Table 2).

Second, vascular stiffness was also significantly reduced within 1 month of switching in smokers of ≤20 pack-years compared with in those who smoked >20 pack-years, suggesting that the trend toward lower blood pressure in the EC arms could be important. Longer term studies are required to detect whether there are statistically and clinically significant reductions in blood pressure when switching from TC to EC as a result of improvements in vascular stiffness.

Third, switching to EC from TC may benefit females more than males and this is also seen in females who were less compliant (dual use). However, this was a subgroup analysis of our data and should be interpreted with caution. Nevertheless, female smokers face more health risks than male smokers do; they are more likely to develop lung cancer (21) and are almost twice as likely to have a myocardial infarction as a result of their smoking (22). The worrying trend worldwide of increased TC prevalence among women (23) suggests that further measures are urgently required to reduce harms associated with TC. Therefore, the switch to EC may be considered a vascular harms reduction measure for both sexes but particularly for the 200 million women worldwide who currently smoke TC (24).

Fourth, those who complied best with allocated therapy, as indicated by exhaled CO levels, benefitted the most in terms of improvement in endothelial function. Our data shows that those who avoided dual use and had lowest CO levels derived greater vascular benefit from switching. Dual use of EC is a highly prevalent reality worldwide (25). The benefits of total switching may have been even larger if subjects fully complied with the switch. This finding could be used to encourage smokers who dual use to minimize TC exposure.

Finally, there was no difference observed between the 2 EC arms (with and without nicotine) for this short-term study. Early improvement appears to be unrelated to the abstinence from nicotine but rather from other toxic material produced by combustion in TC. Further investigation is required to understand the impact of nicotine itself on longer term vascular function.

In addition to these findings, we found a reduction in resting heart rate in the >20 pack-years cohort who switched to EC. The association between resting heart rate and CV events is well known (26,27) and the link between smoking cessation and reduction in heart rate has been previously demonstrated in other studies (28). However, this present study suggests that a switch from TC to EC might also achieve this early on in chronic smokers. A reduction in resting heart rate as seen in this cohort of high CV risk, chronic, heavy smokers would yield the greatest benefit, further supporting the benefits of these cohorts switching from TC to EC. Whether this might be a transient phenomenon or translates to more sustained benefits requires further investigation.

We stress that whereas this study provides new evidence for the rapid improvement of vascular function when switching from TC to EC and therefore suggests that from a vascular perspective, EC may be a less harmful alternative to TC, there is no justification nor evidence from our work to state that EC are safe per se and therefore should never be viewed by nonsmokers as harmless devices to try.

### Comparison with other studies

The vascular impact of EC is a new and evolving field and as such there remains a significant paucity of research in this area. Carnevale et al. (29) reported a small (n = 40) single-use crossover study that demonstrated that although both TC and EC had unfavorable effects on markers of oxidative stress and FMD, EC had a lesser impact than TC did. This result of our present study is consistent with this finding. Hajek et al. (16) recently reported that EC was more effective for smoking cessation than nicotine replacement therapy was when both products were accompanied by behavioral support.

### Study limitations

This was a single-center study. We could not perform a full 3-arm randomized controlled design as it was unethical for participants who wished to quit smoking to be allocated to the smoking arm. We created a propensity score as an adjustment covariate in the regression models to allow for any potential bias and the results remained consistent. Baseline characteristics of the cohorts were also comparable. The duration of effect tested was deliberately short as the primary purpose of the study was to investigate whether there were early vascular benefits from switching from TC to EC and the results are reassuring. However, longer follow-up is required to determine whether males also benefit to the same level as females do and whether these changes seen are sustained and to assess the impact of nicotine in EC. There are many different EC devices available in the market, and we tested only 1 device for consistency of effect. Future comparative studies among different devices are required. Finally, we used endothelial dysfunction as an early indicator of CV disease and a surrogate for CV events. However, endothelial dysfunction has consistently been shown to correlate well with longer term CV outcomes (12,13,30).

## Conclusions

Smokers, particularly females, who switch from TC to EC derive significant benefits in terms of vascular health, and this improvement is seen early on. From a vascular health perspective, recommendations of switching from TC to EC could be considered a vascular harms reduction measure. Further investigation is required on the long-term CV and non-CV effects of these devices.

Perspectives **COMPETENCY IN MEDICAL KNOWLEDGE:** Smoking tobacco cigarettes is known to be harmful. In theory, EC contain fewer harmful substances, but the health risks of EC are currently not fully known.

**COMPETENCY IN PATIENT CARE AND PROCEDURAL SKILLS:** Patients who wish to stop smoking TC should be offered less harmful options including switching to EC.

**TRANSLATIONAL OUTLOOK:** This study demonstrates the early vascular impact of switching from TC to EC. Therefore, switching to EC may be considered a vascular harms reduction measure.
