# Supplementary material for: Cardiovascular Effects of Switching From Tobacco Cigarettes to Electronic Cigarettes
Source: J Am Coll Cardiol. 2019 Dec 24;74(25):3112–20. doi: 10.1016/j.jacc.2019.09.067 (PMC6928567; doi:10.1016/j.jacc.2019.09.067)
Supplement: Online Tables 1 and 2 [file mmc1.docx]

**Supplementary Data**

Online Table 1. Variables Used for Propensity Score Adjustment

| **Variables** |
| --- |
| Sex—male, n (%) |
| Age, mean (95% CI) |
| Weekly alcohol intake (units), median (IQR) |
| BMI, mean (95% CI) |
| Employment status, n (%) |
| Systolic BP (mm Hg), mean (95% CI) |
| Diastolic BP (mm Hg), mean (95% CI) |
| Heart rate (bpm), mean (95% CI) |
| CO% COHb, median (IQR) |
| Oxidized LDL mU/L, median (IQR) |
| Hs-CRP, mg/L, median (IQR) |
| PAI-1, median (IQR) |
| t-PA, median (IQR) |
| Cuff change (%), mean (95% CI) |
| Average integral % change, median (IQR) |
| Alx@75 (%), median (IQR) |
| Carotid femoral PWV m/s, median (IQR) |
| Years smoked, median (IQR) |
| Any previous use of e-cigarette |
| Other smokers in the home |

Online Table 2. E-Cigarette Compliance: Mean Number of Cigarettes Smoked in Those Who Were Noncompliant (Middle and Upper Tertiles of CO)

|  | **Number of Participants** | **Tobacco Cigarettes/day (mean)** |
| --- | --- | --- |
| E-cigarette with nicotine |  |  |
| Middle tertile CO (6–11 ppm) | 9 | 0.52 |
| Upper tertile CO (12–32ppm) | 10 | 2.14 |
|  |  |  |
| E-cigarette without nicotine |  |  |
| Middle tertile CO (6–11 ppm) | 13 | 3.22 |
| Upper tertile CO (12–32 ppm) | 6 | 10.53 |
